# Semaglutide reduces cardiovascular events regardless of metformin use: a post hoc subgroup analysis of SUSTAIN 6 and PIONEER 6

**DOI:** 10.1186/s12933-022-01489-6

**Published:** 2022-04-28

**Authors:** Mansoor Husain, Agostino Consoli, Alessandra De Remigis, Anna Sina Pettersson Meyer, Søren Rasmussen, Stephen Bain

**Affiliations:** 1 Ted Rogers Centre for Heart Research, Department of Medicine, University of Toronto, Peter Munk Cardiac Centre, Toronto General Hospital, 200 Elizabeth St, PMCRT 3-904, M5G2C4 Toronto, Canada; 2 DMSI & CAST, University G. d’Annunzio, Chieti-Pescara, Italy; 3 Novo Nordisk A/S, Søborg, Denmark; 4 Swansea University Medical School, Swansea, UK

**Keywords:** Semaglutide, SUSTAIN 6, PIONEER 6, Major adverse cardiovascular event, Cardiovascular outcome trial, Metformin

## Abstract

**Background:**

Cardiovascular outcome trials (CVOTs) are conducted on a background of standard of care including metformin. These analyses sought to determine whether the cardiovascular (CV) effects of semaglutide and other glucagon-like peptide-1 receptor agonists (GLP-1RAs) vary according to baseline metformin use.

**Methods:**

A post hoc analysis was conducted using pooled SUSTAIN 6 and PIONEER 6 CVOT data in subjects with and without metformin use at baseline. Additionally, a trial-level meta-analysis was conducted using data from seven CVOTs with GLP-1RAs–SUSTAIN 6, PIONEER 6, HARMONY OUTCOMES, LEADER, REWIND, EXSCEL and AMPLITUDE-O–including adults with type 2 diabetes at high CV risk, and a primary endpoint of time to first major adverse CV event (MACE).

**Results:**

In the post hoc analysis, the no-metformin subgroup was older, with a higher body mass index, lower estimated glomerular filtration rate and higher CV risk at baseline vs the metformin subgroup. Hazard ratios (95% confidence intervals) for the reduction in risk of MACE with semaglutide vs placebo in the metformin and no-metformin subgroups were 0.70 (0.55;0.89) and 0.86 (0.60;1.22), respectively. No significant interaction between the treatment effect on MACE and metformin subgroup was observed. Findings for other CV endpoints were similar. In the meta-analysis, treatment effect (GLP-1RA vs placebo) on CV outcomes was no different with vs without baseline metformin (overall ratio between the hazard ratios for metformin vs no-metformin 1.09 [0.96;1.22]).

**Conclusion:**

These findings indicate that the CV outcomes for semaglutide were similar regardless of baseline metformin use, which may also apply to all GLP-1RAs.

*Trial registration* SUSTAIN 6 (NCT01720446), PIONEER 6 (NCT02692716).

**Supplementary Information:**

The online version contains supplementary material available at 10.1186/s12933-022-01489-6.

## Background

Given the increased risk of cardiovascular (CV) morbidity and mortality among people with type 2 diabetes [1], the results of CV outcome trials (CVOTs) that showed a reduction in CV events with some glucagon-like peptide-1 receptor agonists (GLP-1RA) [2–5] and sodium–glucose cotransporter-2 inhibitors (SGLT-2is) [6, 7], have heralded a new era in diabetes management. Based on the findings of such CVOTs, management guidelines have been updated to recommend the use of GLP-1RAs and SGLT-2is with proven CV benefits as first-line therapies in people with type 2 diabetes at high or very high risk of, or with atherosclerotic CV disease [8, 9]. However, because the CVOTs were conducted on a background of standard of care [2–7], the majority of trial participants were receiving concomitant metformin. This raises the question of whether the CV benefits of GLP-1RAs and SGLT-2is are contingent on background metformin use, or whether they are also observed in patients not receiving metformin; the question is salient given that guideline updates from the European Society of Cardiology/European Association for the Study of Diabetes and the American Diabetes Association recommend these therapies as first-line agents in the treatment of drug-naïve patients with type 2 diabetes and high or very high risk of a CV event [8, 9]. Recent post hoc and exploratory subgroup analyses of trials with the GLP-1RAs liraglutide and dulaglutide and the SGLT-2is empagliflozin and dapagliflozin have examined this and have suggested that the CV benefits of these agents are observed independent of background metformin use [10–13]. Furthermore, the use of metformin and the CV effects of glucose-lowering agents, including some GLP-1RAs (liraglutide and albiglutide), SGLT-2is and dipeptidyl peptidase-4 inhibitors (DPP-4i), have been investigated in meta-analyses, which have shown no evidence that metformin use at baseline modified the CV effects of GLP-1RAs or SGLT-2is [14, 15].

As semaglutide is a GLP-1RA with proven CV benefits, the aim of this post hoc analysis was to determine whether its effect on CV events varies according to metformin use. To make as robust an examination as possible, the analysis was conducted using data from both SUSTAIN 6 [4] and PIONEER 6 [16], which were pre-approval CVOTs of subcutaneous and oral semaglutide, respectively, and which had similar designs and subject populations. Additionally, a comprehensive meta-analysis of published GLP-1RA CVOTs with subgroup data available was conducted. The aim of the analysis was to establish the effect of metformin use at baseline on the CV effects of GLP-1RAs as a drug class.

## Methods

### Trial designs

The trial designs for SUSTAIN 6, PIONEER 6, HARMONY OUTCOMES, LEADER, REWIND, EXSCEL and AMPLITUDE-O have been reported previously [2–5, 16–18]. In brief, adults with type 2 diabetes at high risk of a CV event were randomized to a GLP-1RA or placebo in addition to standard of care. The primary endpoint in all trials was time to first major adverse CV event (MACE; a composite of CV death, non-fatal myocardial infarction [MI] or non-fatal stroke). GLP-1RAs by trial were as follows: SUSTAIN 6, semaglutide (once-weekly, subcutaneous); PIONEER 6, semaglutide (daily, oral); HARMONY OUTCOMES, albiglutide (once-weekly, subcutaneous); LEADER, liraglutide (daily, subcutaneous); REWIND, dulaglutide (once-weekly, subcutaneous); EXSCEL, exenatide (once-weekly, subcutaneous); AMPLITUDE-O, efpeglenatide (once-weekly, subcutaneous). All trials were approved by Independent Ethics Committees and Institutional Review Boards at each participating center and conducted in compliance with the International Conference on Harmonisation Good Clinical Practice guidelines and the Declaration of Helsinki. All subjects provided written informed consent before any trial-related activities.

### Subgroups, CV risk score and outcomes

In the post hoc analysis of two subgroups (subjects with metformin use and subjects without metformin use at baseline [metformin/no-metformin]), patient-level data from the SUSTAIN 6 and PIONEER 6 trials were pooled: semaglutide (subcutaneous and oral) vs placebo. A CV risk score for each metformin subgroup was calculated based on a previously published CV risk model [19]. In this model, low scores indicate lower baseline CV risk and, vice versa, high scores indicate higher CV risk.

Event adjudication committee-confirmed CV outcomes were assessed in the two metformin subgroups: time to first MACE (composite of three-components: CV death, non-fatal MI or non-fatal stroke)—the primary outcome; time to first expanded MACE (included CV death, non-fatal MI, non-fatal stroke, hospitalization for unstable angina or heart failure and, for SUSTAIN 6 only, revascularization [coronary or peripheral]); death from CV or all causes; time to first hospitalization for heart failure (HHF).

The metabolic outcomes assessed by subgroup were change in glycated hemoglobin (HbA_1c_) and body weight from baseline to week 80 for SUSTAIN 6 and week 83 for PIONEER 6. The safety outcomes assessed by subgroup were investigator-reported serious adverse events (AEs) and patient-reported severe hypoglycemic episodes.

The use of metformin and CV effects of glucose-lowering agents have been investigated in two recently published meta-analyses; however, these two meta-analyses only included two and four GLP-1RAs, respectively [14, 15]. The patient-level and trial-level meta-analysis conducted here includes data from seven CVOTs with GLP-1RAs: SUSTAIN 6, PIONEER 6, HARMONY OUTCOMES, LEADER, REWIND, EXSCEL and AMPLITUDE-O (data required were not reported for ELIXA). Conducted by trial, data from each of the seven trials were stratified into two subgroups, subjects receiving metformin at baseline and subjects with no metformin at baseline (metformin/no-metformin), to evaluate the differences in treatment effect on CV outcomes according to baseline metformin use.

### Statistical analysis

Hazard ratios (HRs; semaglutide:placebo) and 95% confidence intervals (CIs) for CV outcomes were estimated using a Cox proportional hazards model with treatment by metformin subgroup as a fixed factors, stratified by trial (SUSTAIN 6/PIONEER 6) and CV risk at screening (established CV disease and/or chronic kidney disease, or CV risk factors only) (unadjusted analysis). An adjusted analysis was then performed using this model by inclusion of CV–renal risk factors at baseline. Consistency of the treatment effect across the subgroups was assessed using interaction *P*-values, which indicate whether the average treatment effect in a subgroup is significantly different from that in another subgroup, with *P*_interaction_ < 0.05 indicating a statistically significant difference. No adjustment for multiplicity was performed.

To assess the impact of post-randomization changes in metformin use, two sensitivity analyses were conducted for the CV outcomes: (1) censoring for initiation and discontinuation of metformin during the study using the Cox proportional hazards model described above; (2) time-dependent Cox regression with metformin use during trial as a time-dependent variable.

A subgroup analysis using inverse probability weighting was also performed based on a Cox proportional hazards model with treatment (semaglutide, placebo), subgroup and treatment by subgroup interaction as fixed factors, stratified by trial (SUSTAIN 6/PIONEER 6) [20]. Weights were 1/non-stabilized propensity scores (probability) of treatment, derived from logistic regressions for each trial separately with subgroup, baseline covariates and subgroup by baseline covariate interactions. Baseline covariates used were: age, sex, prior cardiovascular event, prior heart failure, diabetes duration, HbA_1c_, estimated glomerular filtration rate (eGFR), weight, body mass index (BMI), diastolic blood pressure, systolic blood pressure, heart rate, low-density lipoprotein (LDL) cholesterol, insulin use, thiazolidinedione use, sulfonylurea use, angiotensin-converting-enzyme inhibitor (ACEi) use, angiotensin II receptor blocker use and statin use.

For change in the metabolic outcomes from baseline to week 80 (SUSTAIN 6)/83 (PIONEER 6), analyses were based on a mixed model for repeated measurements, with treatment by metformin subgroup adjusted for baseline value and trial nested within visits. For the safety analyses, number of events per 100 patient-years were analyzed using a negative binomial regression model with a log link and the logarithm of the observation time (100 years) as offset, with treatment by metformin subgroup as fixed factors adjusted by trial.

All analyses were performed using observed in-trial data from the full analysis set; data used for all analyses (except for the meta-analysis) were patient-level. For CV risk at screening, the P-value for the difference between groups was estimated using a Wilcoxon test. For the meta-analysis (which used both patient-level and trial-level data), HRs (active comparator: placebo) and 95% CIs for CV outcomes were estimated using a Cox proportional hazards model. In the meta-analysis, HRs from the individual trials were pooled according to metformin use at baseline using the fixed- and random-effect method. The HRs were obtained from published papers, except for SUSTAIN 6 and PIONEER 6, for which patient-level data were used. The fixed-effect model assumed all trials were estimating a common treatment effect; the random-effect model assumed that the underlying treatment effect could vary across trials [21].

## Results

### Baseline characteristics

**Fig. 1:**
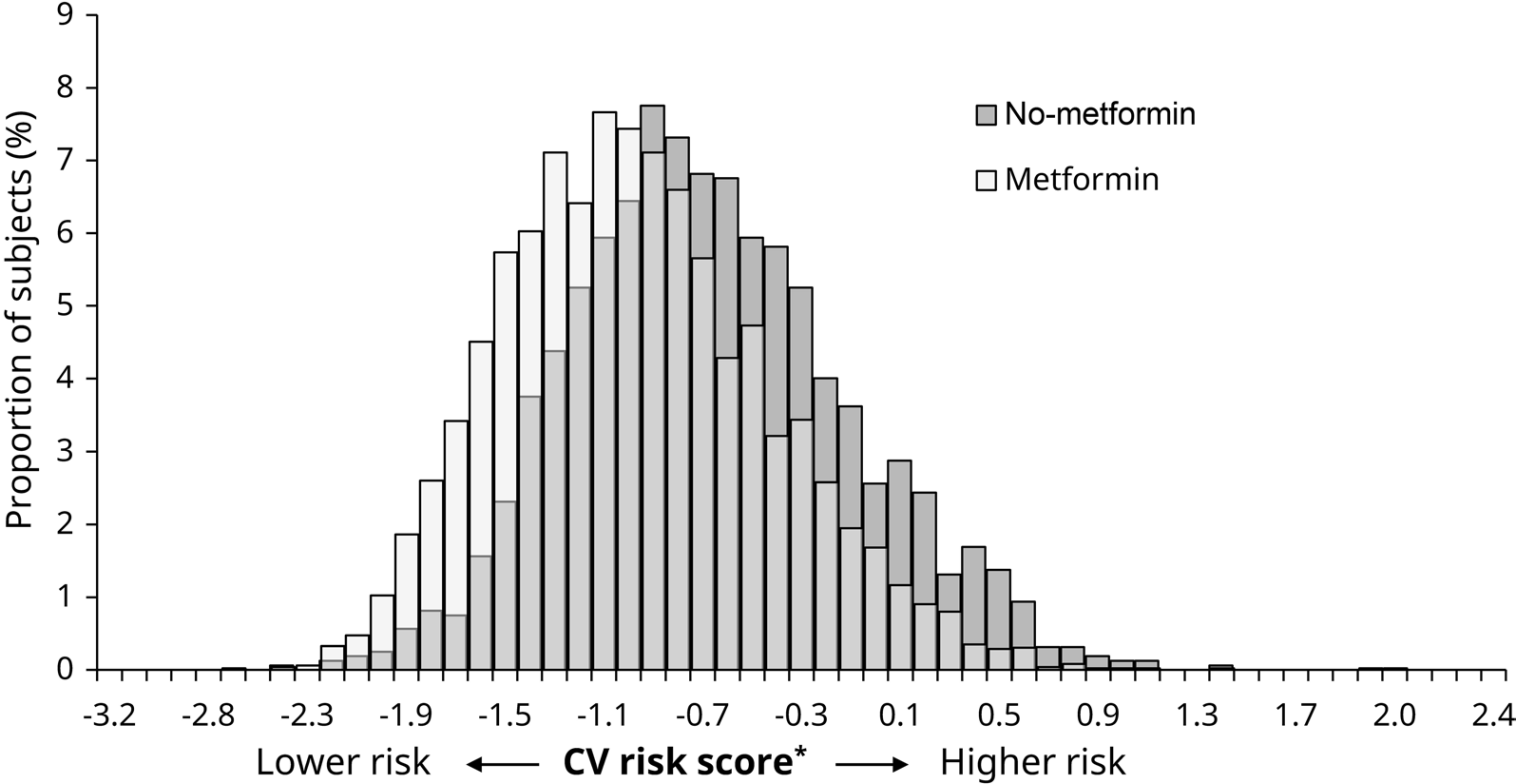
CV risk score^*^ according to metformin use at baseline. The light gray color indicates the overlap between the metformin and no-metformin subgroups. ^*^Derived from a prediction model developed using independent data sets from the liraglutide CV outcomes trial and previously applied to SUSTAIN 6 and PIONEER 6 [19]. *CV* cardiovascular

SUSTAIN 6 and PIONEER 6 included 6,480 subjects, of whom 4,881 (75%) were receiving metformin at baseline. There were notable differences in some baseline characteristics between the subgroups (Table 1). Compared with subjects receiving metformin at baseline, those not receiving metformin at baseline were older (mean [standard deviation (SD)]: 67.1 [7.9] vs 64.8 [7.0] years of age), with higher BMI (33.2 [7.0] vs 32.3 [6.1] kg/m^2^) and lower eGFR (61.9 vs 79.4 mL/min/1.73 m^2^). The no-metformin subgroup also had a higher proportion of subjects with concomitant insulin use (70.7% vs 48.2%) and a lower proportion of subjects with concomitant sulfonylurea use (28.7% vs 40.5%). Additionally, compared with subjects receiving metformin at baseline, the subgroup not receiving metformin had a higher CV risk score at screening (‒ 0.7 [0.6] and ‒ 1.0 [0.5], respectively; *P* < 0.0001) (Fig. 1).

**Table 1:** Baseline characteristics by metformin use subgroup, based on pooled SUSTAIN 6 and PIONEER 6 data

	Metformin	No-metformin	*P*-value
Randomized, *N* (%)	4881	1599	
Age, years	64.8 (7.0)	67.1 (7.9)	< 0.0001
Female, *n* (%)	1718 (35.2)	584 (36.5)	0.3366
Prior cardiovascular event, *n* (%)	2190 (44.9)	694 (43.4)	0.3060
Prior heart failure, *n* (%)	852 (17.5)	313 (19.6)	0.0555
Diabetes duration, years	13.8 (8.0)	16.0 (9.2)	< 0.0001
HbA_1c_, %	8.4 (1.5)	8.6 (1.6)	< 0.0001
HbA_1c_, mmol/mol	68.1 (16.8)	70.5 (17.6)	< 0.0001
eGFR, mL/min/1.73 m^2^	79.4 (19.5)	61.9 (23.8)	< 0.0001
eGFR < 60 mL/min/1.73m^2^, *n* (%)	880 (18.0)	819 (51.2)	< 0.0001
Albuminuria, *n* (%)^a^	855 (36.0)	449 (51.9)	N/A
Concomitant insulin use, *n* (%)	2351 (48.2)	1130 (70.7)	< 0.0001
TZD use, *n* (%)	129 (2.6)	65 (4.1)	< 0.0001^b^
Sulfonylurea, *n* (%)	1978 (40.5)	459 (28.7)	< 0.0001
DPP-4i, *n* (%)	5 (0.1)	2 (0.1)	< 0.0001
Body weight, kg	90.8 (20.3)	93.6 (22.5)	0.0002
BMI, kg/m^2^	32.3 (6.1)	33.2 (7.0)	0.0004
Diastolic BP, mmHg	76.8 (9.9)	75.7 (10.4)	< 0.0001
Systolic BP, mmHg	135.4 (17.1)	136.2 (18.2)	0.1289
Heart rate, bpm	71.8 (11.2)	70.7 (10.9)	0.0015
LDL-cholesterol, mmol/L	2.2 (0.9)	2.4 (0.9)	< 0.0001
LDL-cholesterol, mg/dL	85.6 (35.2)	91.7 (36.1)	< 0.0001
ACEis, *n* (%)	2304 (47.2)	680 (42.5)	0.3541^c^
ARBs, *n* (%)	1800 (36.9)	570 (35.6)	0.3541^c^
Statin, *n* (%)	3798 (77.8)	1200 (75.0)	0.0229^d^

Data are mean (SD) unless stated otherwise

^a^Albuminuria percentage is calculated based on number of subjects with a urinary albumin-to-creatinine ratio measurement at baseline (only available for SUSTAIN 6)

^b^P-value for interaction between different blood glucose-lowering medication, which included TZDs and other classes of medications

^c^P-value for interaction between different anti-hypertensive therapy, which included ACEis, ARBs and other classes of medications

^d^P-value for interaction between different lipid-lowering medications, which included statins and other classes of medications

*ACEi* angiotensin-converting enzyme inhibitor, *ARB* angiotensin receptor blocker, *BMI* body mass index, *BP* blood pressure, *DPP-4i* dipeptidyl peptidase-4 inhibitor, *eGFR* estimated glomerular filtration rate, *HbA*_*1c*_ glycated hemoglobin, *LDL* low-density lipoprotein, *N/A* not available, *SD* standard deviation, *TZD* thiazolidinedione

### CV outcomes and all-cause mortality

**Fig. 2:**
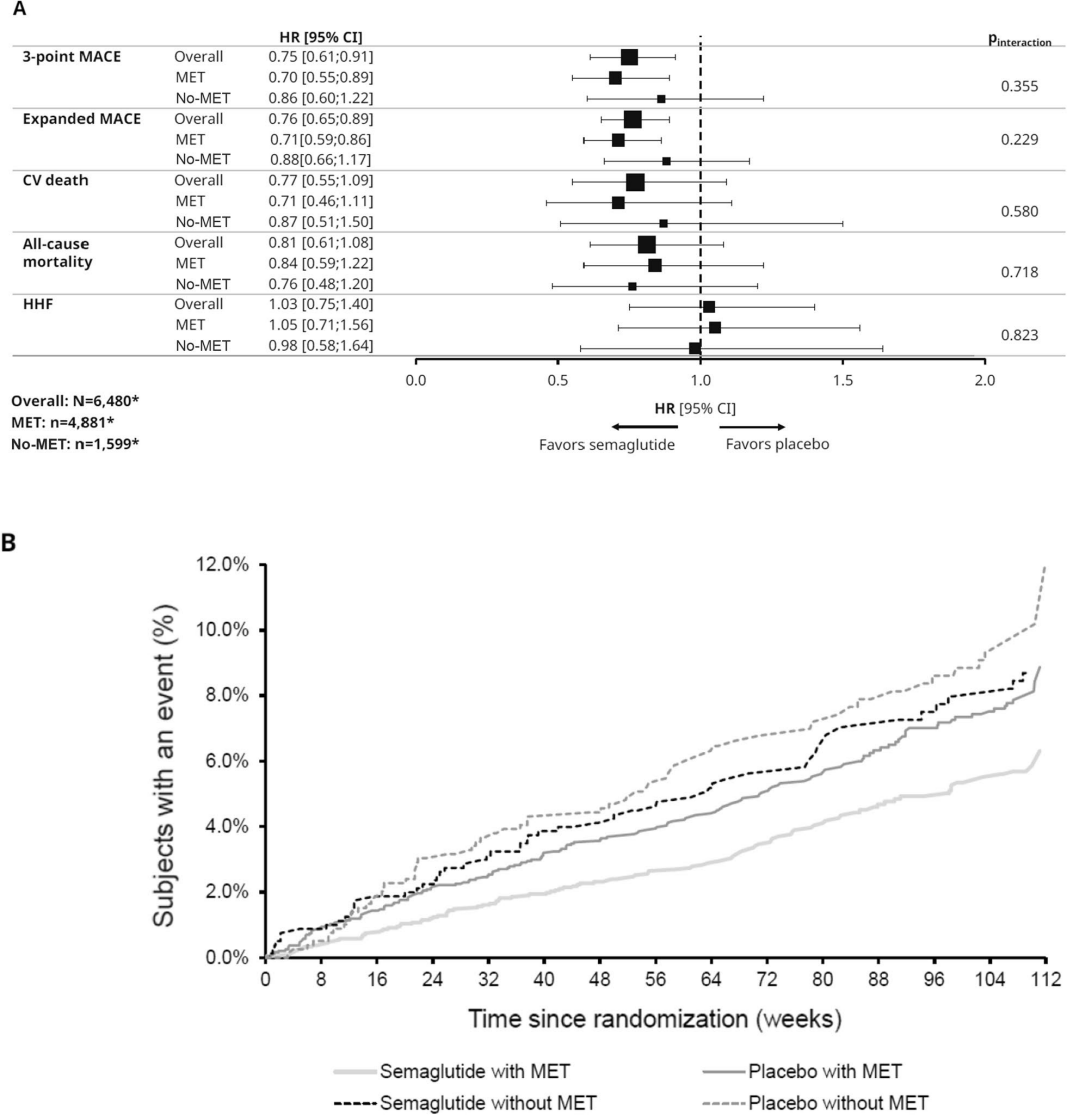
**A** CV outcomes/mortality by baseline metformin use (adjusted analysis); **B** Kaplan–Meier curves of primary outcome by baseline metformin. Figures (**A**) and (**B**) compare semaglutide with placebo. ^*^n-numbers are based on the FAS; the number of subjects included for each endpoint analysis differed according to data availability. **A** Analyses for SUSTAIN 6 and PIONEER 6 are based on a Cox proportional hazards model with treatment (semaglutide, placebo) by metformin subgroup as fixed factors, stratified by trial and CV risk group (established CVD and/or CKD vs risk factors), and adjusted by baseline variables: sex (male vs female), smoker (current smoker, previous smoker, never smoked), previous MI/stroke/TIA (yes vs no), region (European Union, North America, other), antidiabetic treatment (yes vs no), diabetes duration, eGFR-MDRD and age. *CI* confidence interval, *CKD* chronic kidney disease, *CV* cardiovascular, *CVD* cardiovascular disease, *eGFR-MDRD* estimated glomerular filtration rate-modification of diet in renal disease, *FAS* full analysis set, *HbA*_*1c*_ glycated hemoglobin, *HHF* hospitalization for heart failure, *HR* hazard ratio (semaglutide vs placebo), *MACE* major adverse cardiovascular event, *MET* metformin, *MI* myocardial infarction, *TIA* transient ischemic attack

The HRs (95% CIs) for the reduction in risk of three-component MACE (primary endpoint) with semaglutide vs placebo in the metformin and no-metformin subgroups were 0.70 (0.55;0.89) and 0.86 (0.60;1.22), respectively, in the adjusted analysis (Fig. 2). There was no significant interaction between the treatment effect (semaglutide vs placebo) on MACE and subgroup (metformin, yes/no) (*P*_interaction_ not significant). Findings for expanded MACE were similar, both within each subgroup for semaglutide vs placebo and for interaction analyses between the two subgroups (Fig. 2). The results of the unadjusted analyses were similar to the adjusted analyses (Additional file 1: Fig. S1). Additionally, there was no significant reduction in the HR for CV death, all-cause mortality or HHF in either metformin subgroup and no significant interaction between the treatment effect (semaglutide vs placebo) on these outcomes and subgroup (metformin, yes/no) (*P*_interaction_ not significant for either outcome). Both sensitivity analyses and CV outcomes by trial supported the findings of the main analysis (Additional file 1: Figs. S2–5). The results of the propensity analysis also supported the main analysis (Additional file 1: Fig. S6).

### Metabolic outcomes

**Fig. 3:**
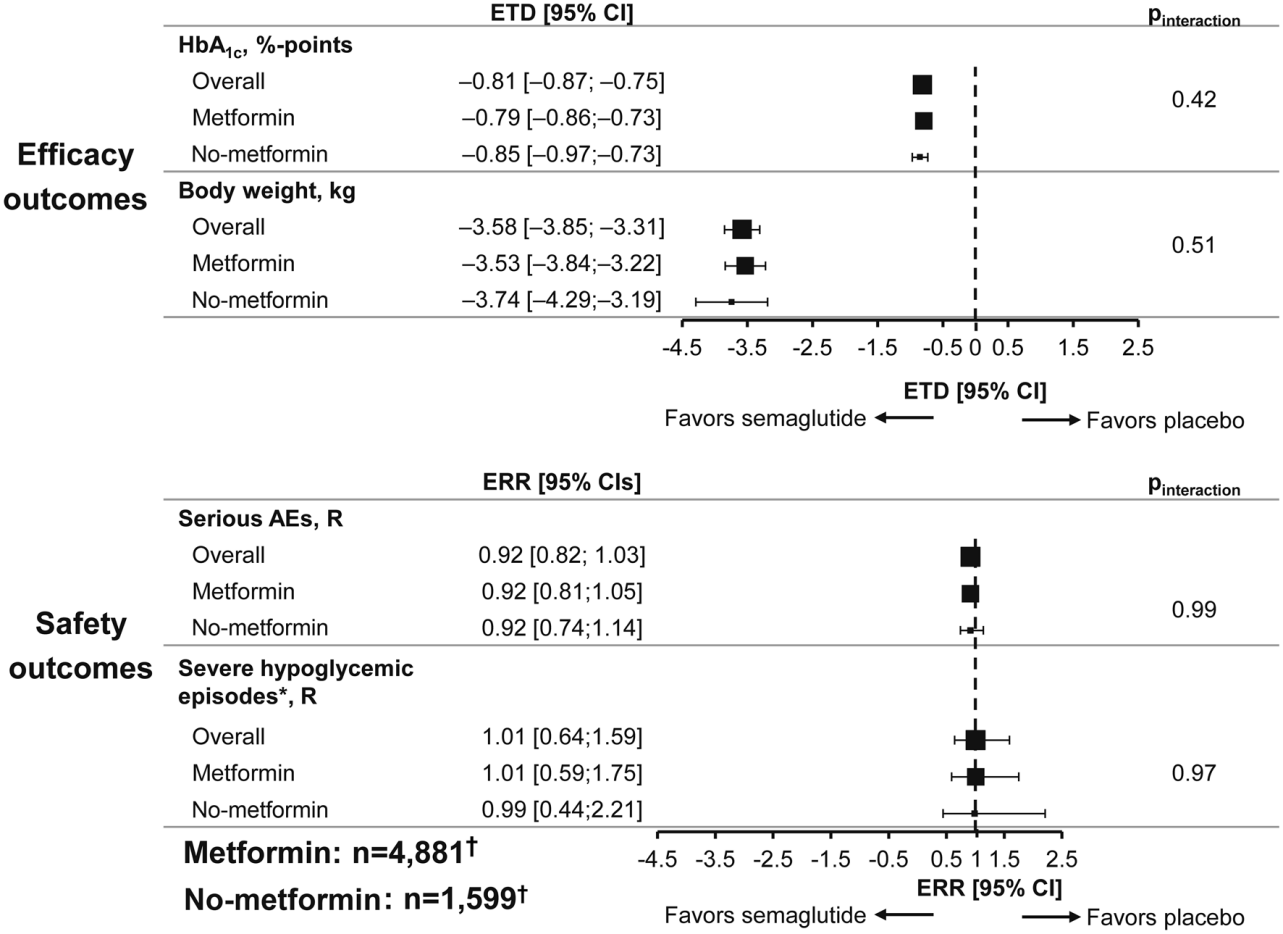
Metabolic and safety outcomes with semaglutide vs placebo by baseline metformin use. Figure compares semaglutide with placebo. Data-points are proportional to subgroup size. For change in metabolic outcomes, analyses were based on a mixed model for repeated measurements at week 80 (SUSTAIN 6) or week 83 (PIONEER 6), with treatment by subgroup adjusted for baseline value and trial nested within visits. For the safety analyses, number of events per 100 patient-years were analyzed using a negative binomial regression model with a log link and the logarithm of the observation time (100 years) as offset, with treatment by subgroup as fixed factors adjusted by trial. Dashed vertical lines represent lines of null effect (0 for ETDs and 1 for ERRs). ETD/ERR is for semaglutide vs placebo. ^*^Defined as an episode requiring assistance of another person to actively administer carbohydrate, glucagon, or to perform other resuscitative actions. ^†^n-numbers are based on the FAS; the number of subjects included for each analysis was smaller according to data availability.  *AE* adverse event, *CI* confidence interval, *ERR* estimated risk ratio, *ETD* estimated treatment difference, *FAS* full analysis set, *HbA*_*1c*_ glycated hemoglobin, *MET* metformin, *R* events per 100 patient-years observed

Semaglutide reduced HbA_1c_ and body weight compared with placebo regardless of baseline metformin use (Fig. 3). No significant differences in change from baseline in HbA_1c_ or body weight were observed between the two subgroups (*P*_interaction_: 0.42 and 0.51, respectively).

### Safety

Within each metformin subgroup, the proportions of subjects experiencing either an investigator-reported serious AE or a patient-reported severe hypoglycemic episode with semaglutide and placebo were comparable. Cardiac disorder events classified in the system organ class were experienced by 8.5% and 9.9% of subjects in the metformin at baseline subgroup receiving semaglutide and placebo, respectively, and by 11.7% and 12.7% of subjects in the subgroup not receiving metformin. Gastrointestinal disorders were experienced by 2.8% and 1.9% in the metformin, and 4.5% and 3.4% of subjects in the no-metformin subgroup receiving semaglutide and placebo, respectively.

With both semaglutide and placebo, the proportion of subjects experiencing serious AEs was higher in those without metformin use at baseline compared with those receiving metformin at baseline: 279/805 (34.7%) and 303/794 (38.2%) without metformin with semaglutide and placebo, respectively, and 610/2,434 (25.1%) and 695/2,447 (28.4%) with metformin. Similarly, a higher proportion of subjects experienced severe hypoglycemic episodes in the no-metformin subgroup compared with the metformin subgroup: 18/805 (2.2%) and 20/794 (2.5%) in no-metformin patients with semaglutide and placebo, respectively, and 33/2,434 (1.4%) and 25/2,447 (1.0%) in the metformin at baseline patients. The estimated treatment differences/estimated risk ratios are shown in Fig. 3 for metabolic and safety outcomes, respectively.

### Meta-analysis

**Fig. 4:**
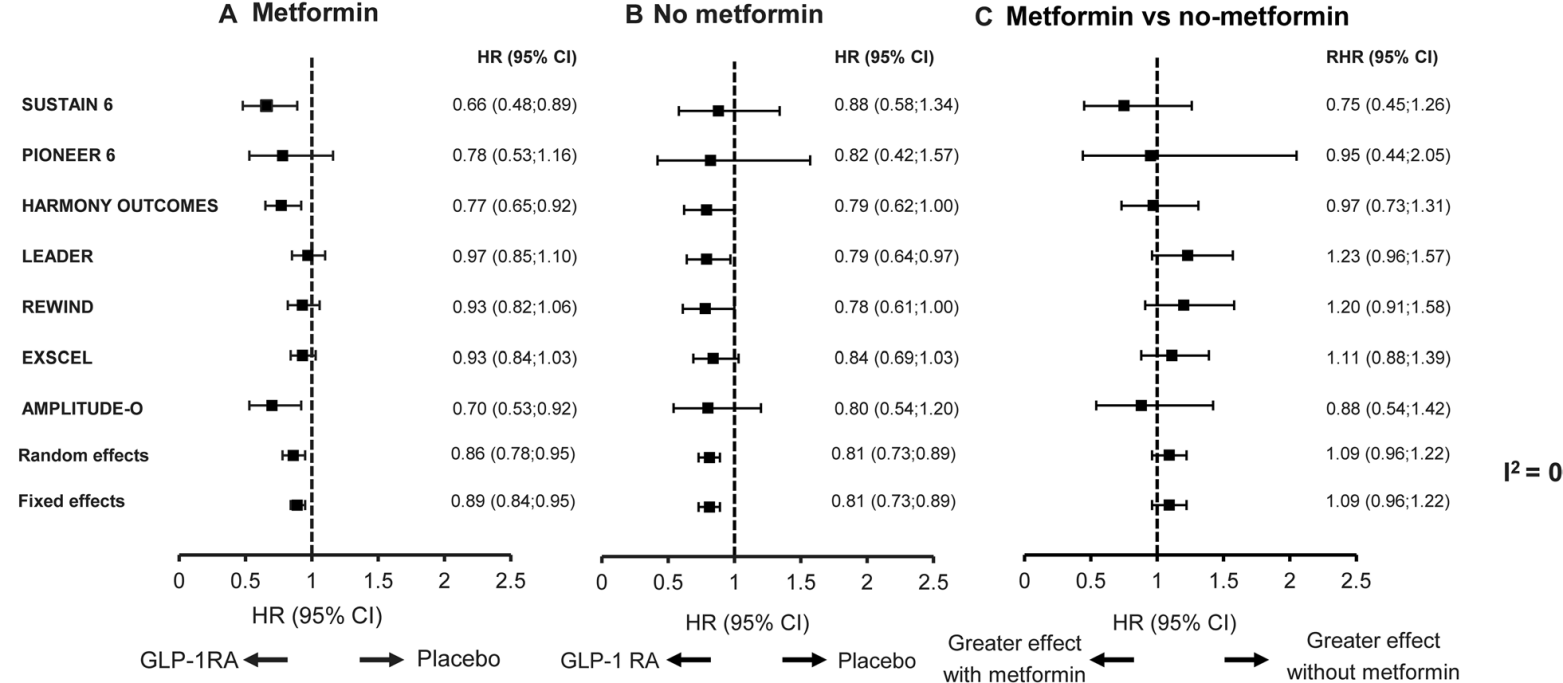
Meta-analysis of effect on MACE by baseline metformin: **A** metformin; **B** no-metformin; **C** metformin vs no-metformin. Figures **A** and **B** compare a GLP-1RA with placebo. Figure C compares the effect of metformin or no-metformin in GLP-1RA trials. Analyses for SUSTAIN 6 and PIONEER 6 are based on a Cox proportional hazards model with treatment (semaglutide, placebo) by metformin subgroup as fixed factors, stratified by trial and CV risk group (established CVD and/or CKD vs risk factors), and adjusted by baseline variables: sex (male vs female), smoker (current, previous, never), previous MI/stroke/TIA (yes vs no), region (European Union, North America, other), antidiabetic treatment (yes vs no), diabetes duration, eGFR-MDRD and age. Trial duration (median follow-up in years) was: 2.1 (SUSTAIN 6); 1.3 (PIONEER 6); 1.6 (HARMONY OUTCOMES); 3.8 (LEADER); 5.4 (REWIND); 3.2 (EXSCEL); and 1.8 (AMPLITUDE-O). **A** includes patients receiving metformin; **B** includes patients not receiving metformin; and **C** includes the ratio between the hazard ratios for the two different groups, metformin and no-metformin. The fixed-effect model assumes all trials are estimating a common treatment effect; the random-effect model assumes the observed estimates of treatment effect can vary across trials. *CI* confidence interval, *CKD* chronic kidney disease, *CV* cardiovascular, *CVD* cardiovascular disease, *eGFR* estimated glomerular filtration rate. *GLP-1RA* glucagon-like peptide-1 receptor agonist, *HR *hazard ratio (GLP-1RA vs placebo), *I*^*2*^ inconsistency across estimates, *MACE* major adverse cardiovascular event, *MDRD* Modification of Diet in Renal Disease, *MI* myocardial infarction, *RHR* ratio between the hazard ratios in metformin vs no-metformin groups, *TIA* transient ischemic attack

Across seven of the CVOTs—SUSTAIN 6, PIONEER 6, HARMONY OUTCOMES, LEADER, REWIND, EXSCEL and AMPLITUDE-O—treatment effect on CV outcomes (GLP-1RA vs placebo) was no different in subjects with or without metformin at baseline (Fig. 4). Although the effect on MACE with and without metformin trended in different directions comparing the ratio of the HR in the different CVOTs, there was no statistical evidence to suggest that baseline metformin use modified the CV effect of any of the GLP-1RAs (Fig. 4). The random-effect estimated ratio between the HRs for metformin vs no-metformin groups was similar (1.09 [0.96;1.22], *P* = 0.3921), and inconsistency across the estimates was 0 (I^2^), suggesting no heterogeneity was observed between trials. Similar results were also obtained using a fixed-effect model.

## Discussion

This analysis indicates that semaglutide provides CV benefits regardless of baseline metformin use, as there was no significant interaction between the semaglutide treatment effect and metformin use at baseline. This finding was consistent across the unadjusted and adjusted analysis and was supported by both sensitivity analyses and the propensity analysis.

For metabolic outcomes, there was homogeneity in the treatment differences across the metformin subgroups.

More serious AEs and severe hypoglycemic episodes were observed in subjects not receiving metformin than in those receiving metformin at baseline. One hypothesis that may explain the safety findings in this analysis is that metformin tends to be discontinued in more frail individuals, such as the elderly and those with decreased kidney function [22]. In these CVOTs, patients without metformin use at baseline may represent a frailer subgroup, who are at greater risk of serious AEs, than the metformin subgroup. Clinically meaningful differences in their baseline characteristics support this hypothesis, as does the externally derived CV risk score.

Compared with the metformin subgroup, those without metformin at baseline were also more likely to be receiving concomitant insulin at baseline, which may help to explain the increased proportion of subjects in the no-metformin subgroup who experienced a severe hypoglycemic episode. The no-metformin subgroup may also have been at increased risk of hypoglycemia, as subjects in this group were older and with a lower eGFR. Although the risk of hypoglycemia with GLP-1RAs is low due to their glucose-dependent mechanism of action [23], when used in combination with other drugs the risk of hypoglycemia may increase. Indeed, lowering the dose of insulin or sulfonylurea can reduce this risk when initiating treatment with semaglutide [24].

The meta-analysis showed that metformin use did not modify the CV effects in any of the trials analyzed. These results support the findings of two similar meta-analyses that looked at the effect of baseline metformin use in the HARMONY-OUTCOMES and LEADER trials, and the HARMONY-OUTCOMES, REWIND, EXSCEL and LEADER trials, respectively [14, 15], as well as individual analyses investigating the impact of baseline metformin use on CV risk in patients treated with different GLP-1RAs reported previously [10–12]. This includes the recently reported AMPLITUDE-O analyses, which suggested that the CV benefits seen with efpeglenatide were independent of baseline metformin use [17]. The results are also supported by a meta-analysis considering only data from subjects not receiving metformin at baseline in the HARMONY-OUTCOMES and LEADER trials, which found that GLP-1RAs are effective at reducing CV risk in metformin-naïve subjects [25].

While a full discussion of the mechanisms behind GLP-1RA-mediated CV risk reduction is beyond the scope of this article, we note that the interaction P-values suggest no difference in the treatment effect on CV outcomes when semaglutide is used with or without metformin. This finding indicates that, while glycemic control may play a role in CV risk reduction, it is not the only mechanism involved. This is in line with previous findings with semaglutide and other GLP-1RAs [26].

Limitations of both the analysis reported here and previous meta-analyses are that the trials they include were not specifically designed to address these research questions, and that the trials included subjects at high risk of a CV event and, as such, may not be representative of the broader type 2 diabetes population. Additionally, one impetus for this analysis was the updates to guidelines stating that GLP-1RAs can be used as first-line therapy in patients with type 2 diabetes and high CV risk [8, 9]. First-line therapy indicates use in treatment-naïve patients; however, the no-metformin subgroup is not precisely representative of such a population. Also, there are limitations inherent to any post hoc (vs prospective) analyses of randomized clinical trials. A further limitation of the present post hoc analysis of SUSTAIN 6 and PIONEER 6 is that the number of CV events was low, and the trial durations were shorter than for other CVOTs; therefore, exposure to study drug was limited. There were also fewer subjects in the no-metformin subgroup compared with the metformin subgroup, and the interaction results have limited statistical power; furthermore, the test for interaction did not indicate the direction of the treatment effect. Additionally, although an adjusted analysis was conducted, not all factors may have been accounted for. Urinary albumin-creatinine ratio was, for instance, not included as these data were not available from PIONEER 6. However, a subgroup analysis using inverse probability weighting including a wider range of covariates was conducted, and the findings of this analysis were aligned with those of the main analysis. The possible effect of other glucose-lowering medications was also not analyzed. The trials included in the meta-analysis had different durations; subject characteristics may also have differed between trials and between the subgroups with and without metformin, which could influence the CV effect of different drugs.

Despite these limitations, the data reported here add to the growing body of evidence that the effect of semaglutide on MACE is similar regardless of metformin use at baseline. Our meta-analysis showed that baseline metformin did not modify the CV benefit shown across CVOTs evaluating different GLP-1RA therapies. While, for reasons of cost and access, metformin may be used as first-line therapy in some countries, the data presented here and elsewhere support the use of GLP-1RAs, such as semaglutide, in patients with type 2 diabetes and a high or very high risk of a CV event whether they are receiving metformin or not.

## Conclusions

In conclusion, these findings indicate that the CV, metabolic and safety outcomes for semaglutide were similar regardless of metformin use at baseline. These findings may be applicable to all GLP-1RAs, and support the guideline recommendation that GLP-1RAs such as semaglutide should be used in patients with type 2 diabetes and a high or very high risk of CV events [8, 9].

## Supplementary Information

**Additional file 1: Figure S1.** CV outcomes by metformin use at baseline (unadjusted analysis). **Figure S2.** CV outcomes by metformin use at baseline censored for initiation and discontinuation of metformin. **Figure S3.** CV outcomes by metformin use at baseline adjusted for time-dependent metformin use during trial (yes/no). **Figure S4.** CV outcomes and all-cause mortality with semaglutide* vs placebo by baseline metformin use (SUSTAIN 6). **Figure S5.** CV outcomes and all-cause mortality with semaglutide* vs placebo by baseline metformin use (PIONEER 6). **Figure S6.** CV outcomes and mortality by baseline metformin use using inverse probability weighting.

## Data Availability

Will individual participant data be available (including data dictionaries)?: Individual participant data will be shared in data sets in a de-identified/anonymized format. What data in particular will be shared?: Data sets from Novo Nordisk-sponsored clinical research completed after 2001 for product indications approved in both the EU and USA; What other documents will be available?: The study protocol and redacted Clinical Study Report (CSR) will be available according to Novo Nordisk data sharing commitments; When will data be available (start and end dates)?: The data will be available permanently after research completion and approval of product and product use in both EU and USA. No end date; With whom will data be shared?: With *bona fide* researchers submitting a research proposal and requesting access to data; For what types of analyses?: For use as approved by the Independent Review Board (IRB) according to the IRB Charter (see www.novonordisk-trials.com); By what mechanism will data be made available?: The access request proposal form and the access criteria can be found at www.novonordisk-trials.com. The data will be made available on a specialized SAS data platform.

## Abbreviations

ACEi: Angiotensin-converting enzyme inhibitor
AE: Adverse event
ARB: Angiotensin receptor blocker
BMI: Body mass index
BP: Blood pressure
CI: Confidence interval
CKD: Chronic kidney disease
CV: Cardiovascular
CVD: Cardiovascular disease
CVOTs: Cardiovascular outcome trials
DPP-4i: Dipeptidyl peptidase-4 inhibitor
eGFR: Estimated glomerular filtration rate
ERR: Estimated risk ratio
ETD: Estimated treatment difference
FAS: Full analysis set
GLP-1RA: Glucagon-like peptide-1 receptor agonist
HbA_1c_: Glycated hemoglobin
HHF: Hospitalization for heart failure
HR: Hazard ratio
I^2^: Inconsistency across estimates
LDL: Low-density lipoprotein
MACE: Major adverse cardiovascular event
MDRD: Modification of Diet in Renal Disease
MET: Metformin
MI: Myocardial infarction
N/A: Not available
R: Events per 100 patient-years observed
RHR: Ratio between the hazard ratios in metformin vs no-metformin groups
SD: Standard deviation
SGLT-2i: Sodium–glucose cotransporter-2 inhibitor
TIA: Transient ischemic attack
TZD: Thiazolidinedione
