# Supplementary material for: Semaglutide reduces cardiovascular events regardless of metformin use: a post hoc subgroup analysis of SUSTAIN 6 and PIONEER 6
Source: Cardiovasc Diabetol. 2022 Apr 28;21:64. doi: 10.1186/s12933-022-01489-6 (PMC9052629; doi:10.1186/s12933-022-01489-6)
Supplement: Supplementary file 1 — Additional file 1: Figure S1. CV outcomes by metformin use at baseline (unadjusted analysis). Figure S2. CV outcomes by metformin use at baseline censored for initiation and discontinuation of metformin. Figure S3. CV outcomes by metformin use at baseline adjusted for time-dependent metformin use during trial (yes/no). Figure S4. CV outcomes and all-cause mortality with semaglutide* vs placebo by baseline metformin use (SUSTAIN 6). Figure S5. CV outcomes and all-cause mortality with semaglutide* vs placebo by baseline metformin use (PIONEER 6). Figure S6. CV outcomes and mortality by baseline metformin use using inverse probability weighting. [file 12933_2022_1489_MOESM1_ESM.docx]

# Supplementary materials

**Figure S1 —** CV outcomes by metformin use at baseline (unadjusted analysis)


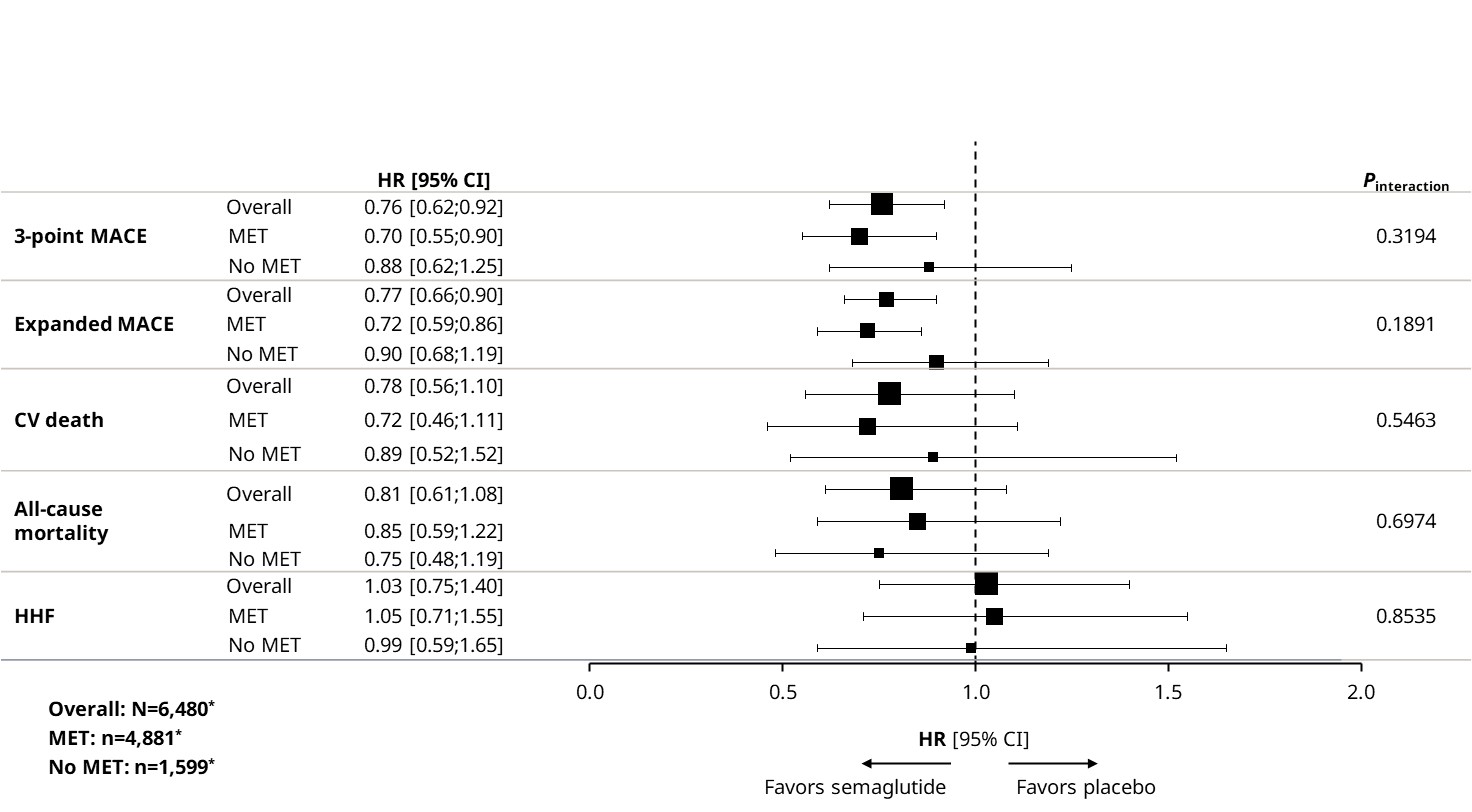


*n values are based on the FAS. Analyses for SUSTAIN 6 and PIONEER 6 are based on a Cox proportional hazards model with treatment (semaglutide, placebo) by MET subgroup as fixed factors, stratified by trial and CV risk group (established CVD and/or CKD vs risk factors). CKD, chronic kidney disease; CV, cardiovascular; CVD, cardiovascular disease; HHF, hospitalization for heart failure; HR, hazard ratio; MACE, major adverse cardiovascular event; MET, metformin.

**Figure S2 —** CV outcomes by metformin use at baseline censored for initiation and discontinuation of metformin


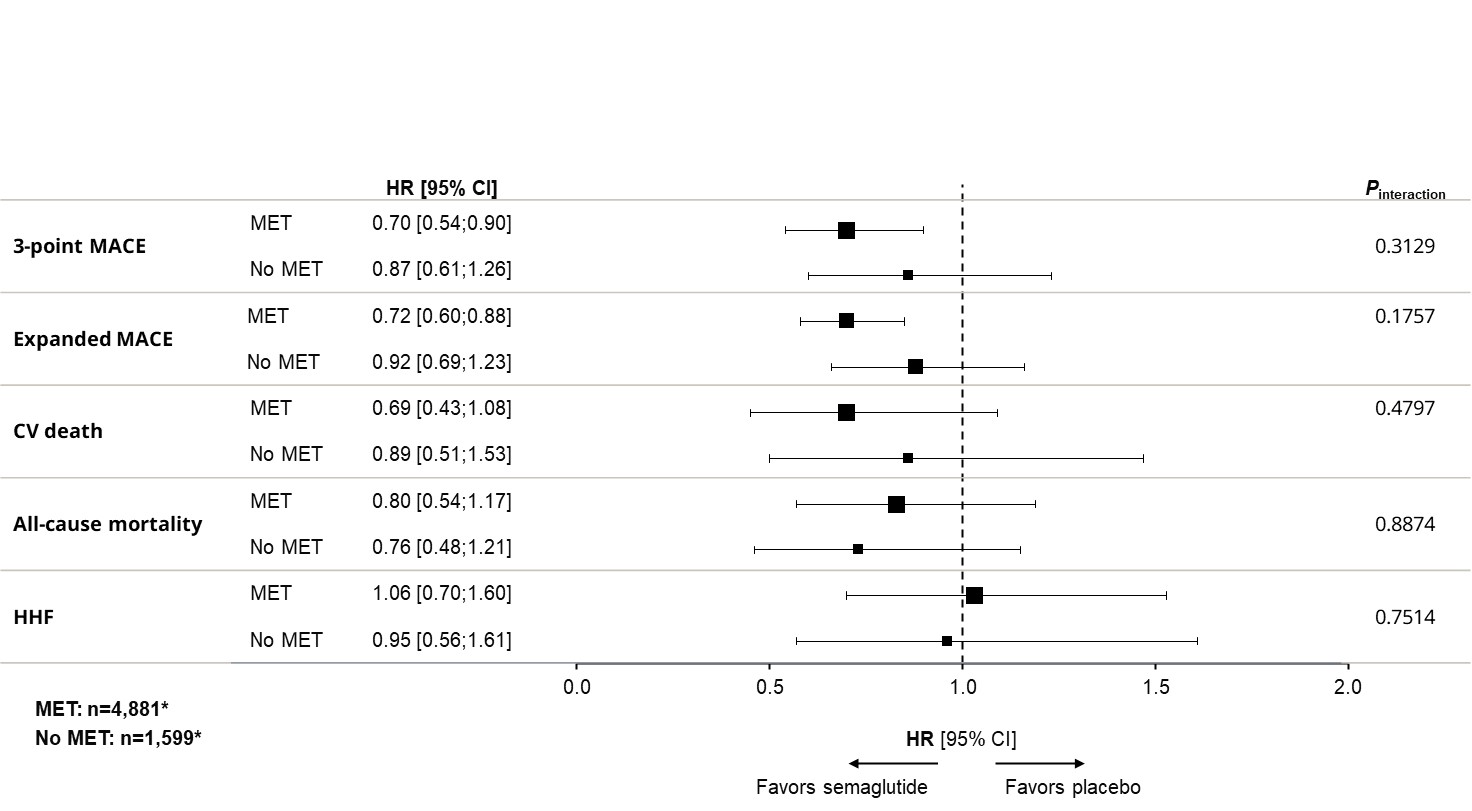


*n values are based on the FAS. Analyses for SUSTAIN 6 and PIONEER 6 are based on a Cox proportional hazards model with treatment (semaglutide, placebo) by MET subgroup as fixed factors, stratified by trial and CV risk group (established CVD and/or CKD vs risk factors). CKD, chronic kidney disease; CV, cardiovascular; CVD, cardiovascular disease; HHF, hospitalization for heart failure; HR, hazard ratio; MACE, major adverse cardiovascular event; MET, metformin.

**Figure S3** **—** CV outcomes by metformin use at baseline adjusted for time-dependent metformin use during trial (yes/no)


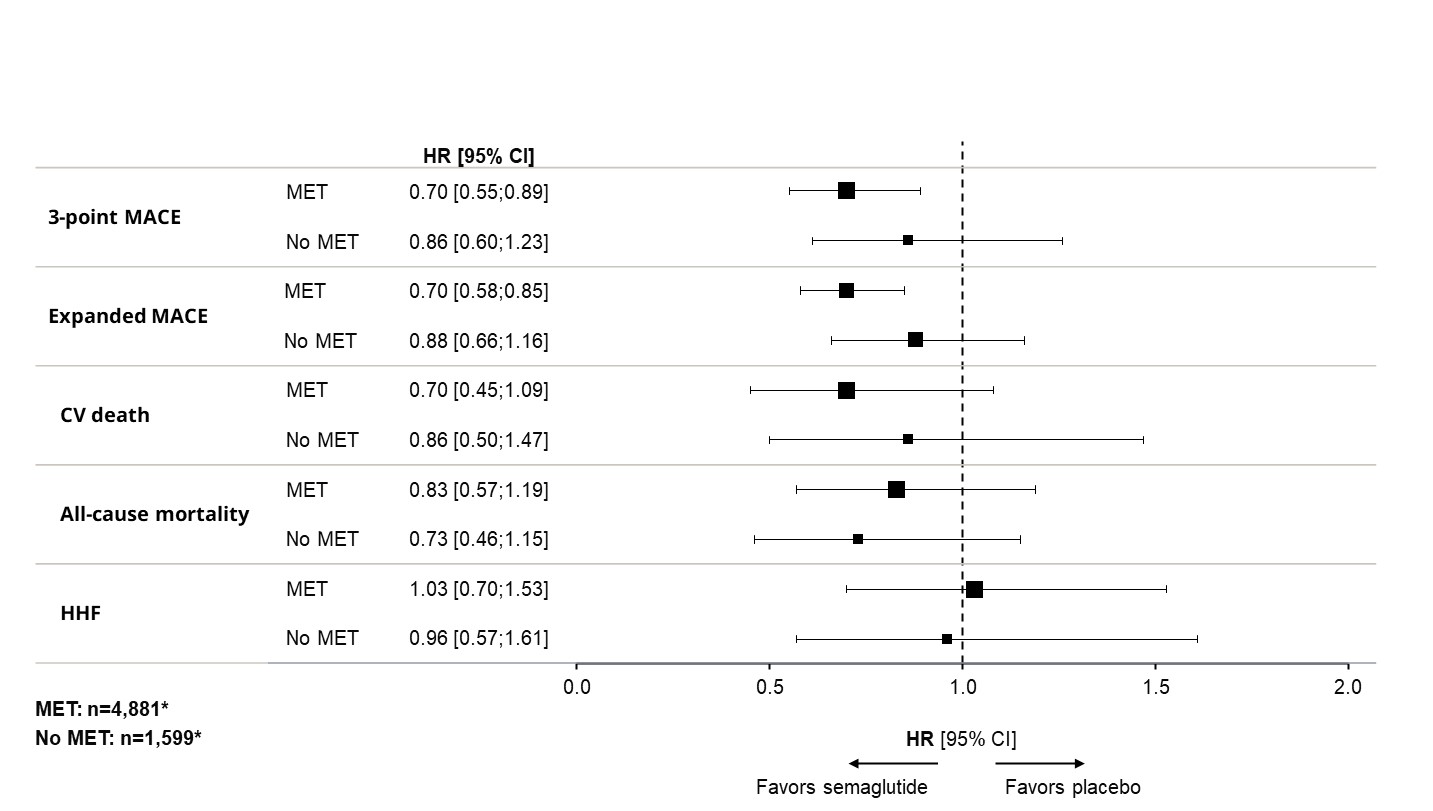


*n values are based on the FAS. Analyses for SUSTAIN 6 and PIONEER 6 are based on a time-dependent Cox proportional hazards model with treatment (semaglutide, placebo) by MET subgroup as fixed factors, stratified by trial and CV risk group (established CVD and/or CKD vs risk factors) and metformin use as a time-dependent factor. CKD, chronic kidney disease; CV, cardiovascular; CVD, cardiovascular disease; HHF, hospitalization for heart failure; HR, hazard ratio; MACE, major adverse cardiovascular event; MET, metformin.

**Figure S4** **—** CV outcomes and all-cause mortality with semaglutide* vs placebo by baseline metformin use (SUSTAIN 6)


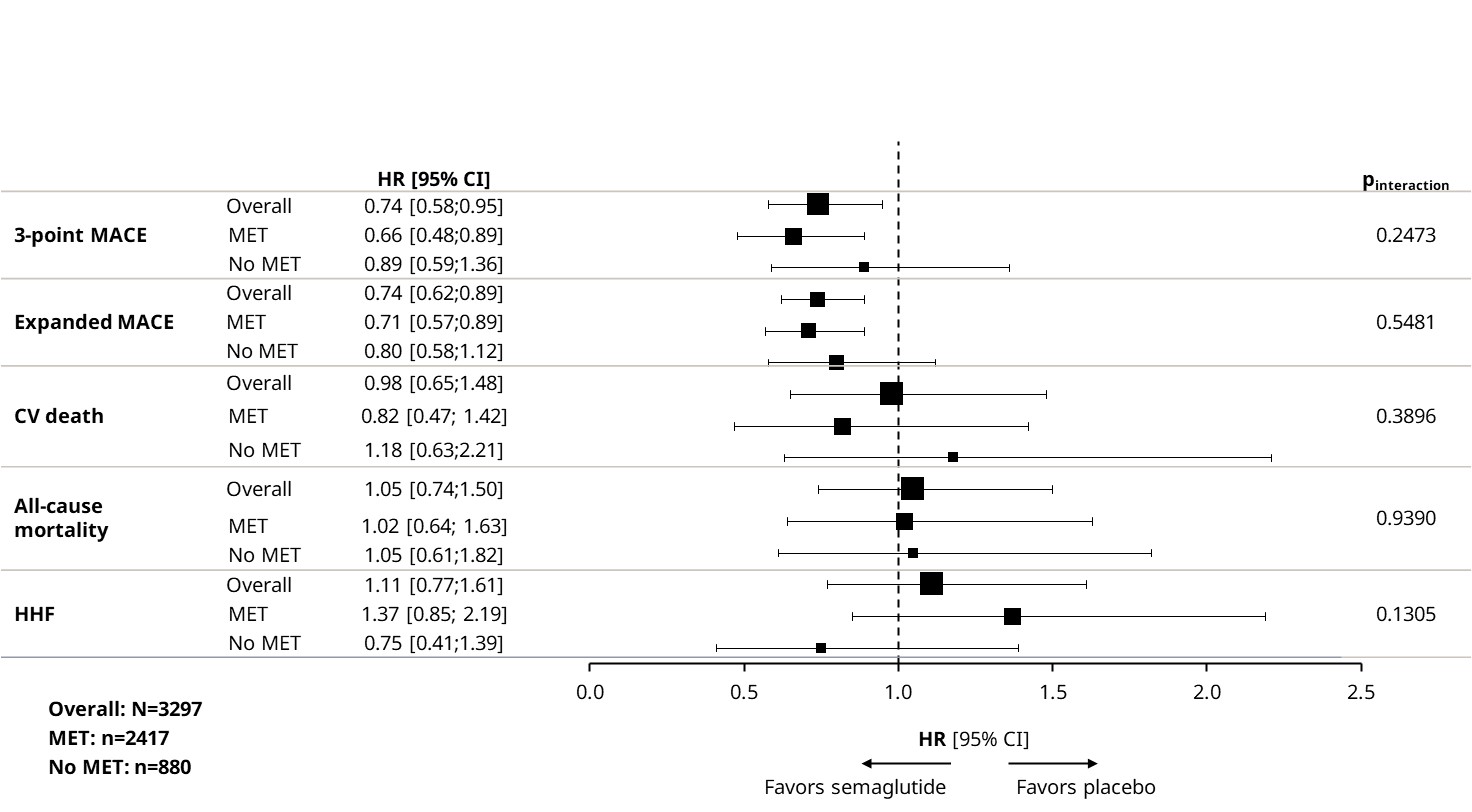


*Once-weekly subcutaneous administration. Analysis is based on a Cox proportional hazards model with treatment (semaglutide, placebo) by MET subgroup as fixed factors. CV, cardiovascular; HHF, hospitalization for heart failure; HR, hazard ratio; MACE, major adverse cardiovascular event; MET, metformin.

**Figure S5** **—** CV outcomes and all-cause mortality with semaglutide* vs placebo by baseline metformin use (PIONEER 6)


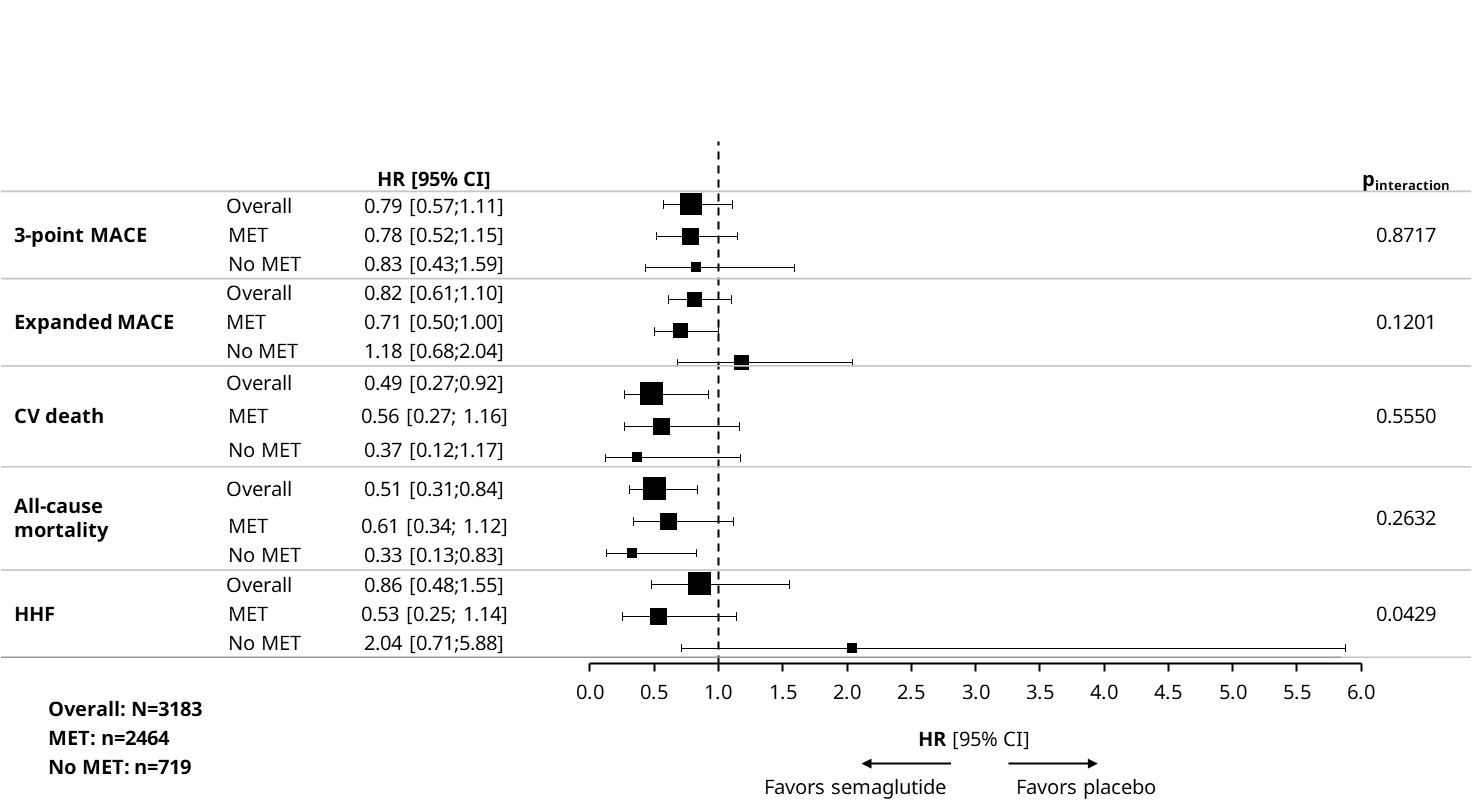


*Oral administration. Analysis is based on a Cox proportional hazards model with treatment (semaglutide, placebo) by subgroup as fixed factors.
CI, confidence interval; CV, cardiovascular; HHF, hospitalization for heart failure; HR, hazard ratio; MACE, major adverse cardiovascular event; MET, metformin.

**Figure S6** **—** CV outcomes and mortality by baseline metformin use using inverse probability weighting


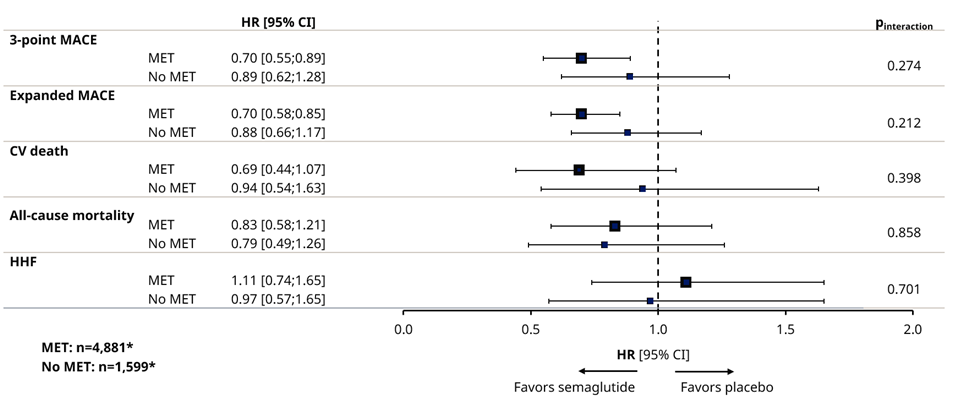


*n-numbers are based on the FAS; the number of subjects included for each endpoint analysis differed according to data availability. The propensity analysis was performed based on a Cox proportional hazards model with treatment (semaglutide, placebo), subgroup and treatment by subgroup interaction as fixed factors, stratified by trial (SUSTAIN 6/PIONEER 6) and using inverse probability weighting. Weights were 1/non-stabilized propensity scores (probability) of treatment, derived from logistic regressions for each trial separately with subgroup, baseline covariates and subgroup by baseline covariate interactions. Baseline covariates used were: age, sex, prior cardiovascular event, prior heart failure, diabetes duration, HbA1c, eGFR, weight, BMI, diastolic blood pressure, systolic blood pressure, heart rate, LDL cholesterol, insulin use, thiazolidinedione use, sulfonylurea use, ACEi use. ACEi, angiotensin-converting-enzyme inhibitor; BMI, body mass index; CI, confidence interval; CV, cardiovascular; eGFR, estimated glomerular filtration rate; FAS, full analysis set; HHF, hospitalization for heart failure; HR, hazard ratio (semaglutide vs placebo); LDL, low-density lipoprotein; MACE, major adverse cardiovascular event; MET, metformin.
